# Treatment Outcomes in Anaplastic Thyroid Cancer

**DOI:** 10.1155/2019/8218949

**Published:** 2019-05-23

**Authors:** Kelsey L. Corrigan, Hannah Williamson, Danielle Elliott Range, Donna Niedzwiecki, David M. Brizel, Yvonne M. Mowery

**Affiliations:** ^1^Duke University School of Medicine, USA; ^2^Department of Biostatistics and Bioinformatics, Duke University Medical Center, USA; ^3^Department of Pathology, Duke University Medical Center, USA; ^4^Department of Radiation Oncology, Duke Cancer Institute, USA; ^5^Department of Surgery, Duke University Medical Center, USA

## Abstract

**Background:**

Anaplastic thyroid cancer (ATC) is rare, accounting for 1-2% of thyroid malignancies. Median survival is only 3-10 months, and the optimal therapeutic approach has not been established. This study aimed to evaluate outcomes in ATC based on treatment modality.

**Methods:**

Retrospective review was performed for patients treated at a single institution between 1990 and 2015. Demographic and clinical covariates were extracted from the medical record. Overall survival (OS) was modeled using Kaplan Meier curves for different treatment modalities. Univariate and multivariate analyses were conducted to assess the relationships between treatment and disease characteristics and OS.

**Results:**

28 patients with ATC were identified (n = 16 female, n = 12 male; n = 22 Caucasian, n = 6 African-American; median age 70.9). Majority presented as Stage IVB (71.4%). Most patients received multimodality therapy. 19 patients underwent local surgical resection. 21 patients received locoregional external beam radiotherapy (EBRT) with a median cumulative dose of 3,000 cGy and median number of fractions of 16. 14 patients received systemic therapy (n = 11 concurrent with EBRT), most commonly doxorubicin (n = 9). 16 patients were never disease free, 11 patients had disease recurrence, and 1 patient had no evidence of disease progression. Median OS was 4 months with 1-year survival of 17.9%. Regression analysis showed that EBRT (HR: 0.174; 95% CI: 0.050–0.613; p=0.007) and surgical resection (HR: 0.198; 95% CI: 0.065–0.598; p=0.004) were associated with improved OS. Administration of chemotherapy was not associated with OS.

**Conclusions:**

Anaplastic thyroid cancer patients receiving EBRT to the thyroid area/neck and/or surgical resection had better OS than patients without these therapies, though selection bias likely contributed to improved outcomes since patients who can undergo these therapies tend to have better performance status. Prognosis remains poor overall, and new therapeutic approaches are needed to improve outcomes.

## 1. Introduction

Thyroid cancer is a prevalent disease that affects 5% of females and 1% of males globally [1]. Anaplastic thyroid cancer (ATC) is the rarest histologic subtype, representing 1-2% of thyroid malignancies with approximately 600 new cases in the U.S. annually [2]. It is the most aggressive type of thyroid cancer and causes significant morbidity and mortality. Older age, male gender, bilateral tumors, presence of local invasion, and/or distant metastasis are unfavorable prognostic factors that are present in the majority of ATC cases [3, 4]. Despite multimodality treatment, outcomes are poor with a median survival of 3-10 months and a 20% 1-year survival rate [5]. Most patients experience tumor location-specific and treatment toxicities including airway compromise, dysphagia, esophagitis, and radiation dermatitis [6].

Optimal management of ATC requires multimodality management by surgeons, radiation oncologists, and medical oncologists. First-line curative treatment requires surgical resection. External beam radiation therapy (EBRT) with chemotherapy is generally employed postoperatively or utilized as definitive therapy for unresectable disease [3, 7]. Commonly used radiotherapy regimens include conventional fractionation (1.5-2 Gy/day) and accelerated twice daily radiation with fraction sizes ranging from 1 to 2 Gy [7]. Radiation treatment volumes vary between comprehensive targeting of the surgical bed, bilateral cervical lymphatics, and anterior mediastinum and more limited coverage of macroscopic disease with margin for microscopic extension. Doxorubicin, commonly combined with cisplatin, has been used for systemic therapy with or without EBRT [8, 9] although other drugs such as paclitaxel have also been utilized [10–12]. Palliative treatment of ATC often involves low-dose radiotherapy directed to the neck or metastatic sites with the intent of mitigating local invasion and associated symptoms [3]. Overall, ATC responds poorly to therapy indicating the need for novel treatment modalities [13].

Few studies have evaluated outcomes and toxicities for the treatment of ATC. Additionally, the efficacy of newer radiation therapy techniques, including intensity modulated radiation treatment (IMRT), has not been widely studied for this disease. Given the limited prospective randomized data and small, heterogeneous retrospective studies of EBRT in this disease, the optimal radiotherapy fractionation regimen and technique remain poorly defined. The purpose of this study was to evaluate treatment regimens, outcomes, and toxicities for ATC. We hypothesized that the administration of locoregional EBRT would improve progression-free and overall survival. We also anticipated that radiotherapy would be well-tolerated, with lower toxicity for patients treated with IMRT compared to conventional 2D or 3D conformal radiation treatment.

## 2. Materials and Methods

### 2.1. Patient Selection

**Figure 1:**
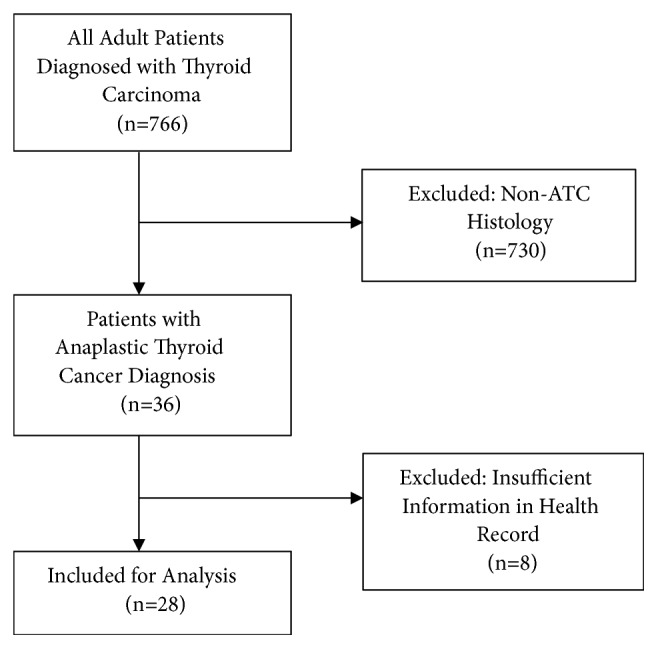
Inclusion criteria.

Records of all adult patients diagnosed with thyroid carcinoma were retrospectively reviewed under an institutional review board-approved protocol at Duke University Medical Center between January 1, 1990, and December 31, 2015. ATC patients who had unavailable electronic and paper health records and patients with nonanaplastic thyroid cancer were excluded (Figure 1).

Patient demographics, disease stage (American Joint Committee on Cancer 8^th^ edition), leukocytosis (WBC ≥ 10,000/mm^3^) at time of ATC diagnosis, histology, pathologic characteristics, receipt and details of surgery, radioactive iodine (RAI) treatment, radiotherapy and/or chemotherapy, results and dates of all imaging studies, status at last follow-up, date(s) of recurrence, treatment-related toxicities, locoregional and distant disease control, progression-free survival, and overall survival were compiled. The following surgical parameters were recorded: surgery type (lobectomy, total thyroidectomy, lymph node dissection, and metastasectomy), margin status, pre- and postoperative thyroid-stimulating hormone (TSH) and thyroglobulin antibodies (TgAb), and toxicity. The following RAI treatment parameters were recorded: RAI uptake, number of courses, and dose of RAI. The following radiotherapy parameters were recorded: technique (intensity modulated radiation therapy [IMRT], 2D or 3D conformal radiation therapy, stereotactic radiosurgery [SRS], stereotactic body radiation therapy [SBRT]), treatment intent (curative versus palliative, defined by treating physician in radiation prescription), radiation site, total dose, schedule, in-field and out-of-field recurrence, and toxicity. Total radiation dose was dichotomized into groups (<4,000 cGy versus ≥4,000 cGy) based on other studies showing significantly improvement outcomes with radiation dose ≥4,000 cGy [14–16]. The following chemotherapy parameters were obtained: drug(s) used, number of courses, number of cycles, and toxicity. All data were entered into a secure REDCap database.

### 2.2. Statistical Analysis

Patient and treatment characteristics were summarized with counts and percentages for categorical variables and with medians and interquartile ranges (IQR) for continuous variables for all patients. Relevant variables specific to receipt of EBRT were also summarized by course, and patient outcomes including recurrence, progression, and cause of death were summarized with counts and percentages.

Overall survival (OS) was modeled using the Kaplan Meier method for different treatment modalities, including dichotomous EBRT vs. no EBRT, surgery vs. no surgery, chemotherapy vs. no chemotherapy, and initial EBRT intent for those who received EBRT. OS was defined as time from ATC diagnosis date to death from any cause, with living patients censored at their date of last assessment. 1-year OS, median OS, and corresponding 95% confidence intervals were presented for all treatment modalities. Recurrence-free survival, defined as time from ATC diagnosis date to first local, regional, or distant recurrence or death from any cause, was also described using Kaplan Meier plots, 1-year survival, and median survival. For recurrence-free survival, patients were censored at their date of last assessment if they did not have any of the specified events.

Cox proportional hazards models were used to assess the univariate and multivariate relationships between OS and selected treatment and patient characteristics. From the univariate model, age, leukocytosis (WBC ≥ 10,000/mm^3^) at time of ATC diagnosis, and receipt of EBRT, surgery, and chemotherapy were chosen as covariates for the multivariate model predicting OS. Only patients with available data were utilized in each model, and effective sample sizes were included in all tables and figures. No adjustments were made for multiple comparisons. All statistical analyses were conducted using SAS version 9.4 (SAS Institute, Cary, NC).

## 3. Results

Of the 766 patients diagnosed with thyroid cancer, 28 patients met inclusion criteria (Figure 1).  Table 1 describes baseline patient characteristics at the time of anaplastic thyroid cancer diagnosis. Patients were predominantly female (57.1%) and Caucasian (78.6%; African-American, 21.4%). Median age at diagnosis was 70.9 (IQR: 63.8–74.7). 12 patients (42.9%) had leukocytosis (WBC ≥ 10,000/mm^3^) at the time of their ATC diagnosis. Six patients had an initial or concomitant diagnosis of differentiated thyroid cancer. A majority of patients presented as Stage IVB disease (71.4%), with remaining stage distribution as follows: 7.1% stage IVA, 17.9% stage IVC, and 3.6% unknown.

Table 2 summarizes surgical, RAI, EBRT, and systemic treatment characteristics. Most patients received multimodality therapy. 2 patients (7.1%) received radioactive iodine treatment after their ATC diagnosis due to a synchronous diagnosis of differentiated thyroid cancer. 19 patients (67.9%) underwent thyroid lobectomy or total thyroidectomy. Of these, one patient required an extensive operation (total laryngopharyngectomy). 5 patients did not have postoperative radiation therapy, including 2 patients intended to have radiation therapy but precluded from further treatment due to postoperative complications requiring tracheostomy placement and subsequent decompensation due to aspiration pneumonia and extensive disease. 15 (53.6%) patients had postoperative radiation therapy. Of these patients, 4 had postoperative airway compromise requiring tracheostomy placement, which delayed radiation therapy in 2 patients. The median time between surgery and radiation therapy was 4 weeks.

21 patients (75.0%) received EBRT to any site, and 19 received locoregional EBRT to the thyroid, thyroid bed, and/or neck. 32.1% of patients had more than one course of EBRT. Of those patients who were treated with EBRT, 66.7% were initially treated with palliative intent and 33.3% were initially treated with curative intent. Initial EBRT course techniques were 2D/3D conformal (n = 7) or IMRT (n = 12), with 2 unknown due to receiving radiotherapy at outside institutions. 14 patients (50.0%) received systemic therapy, 11 of whom had concurrent chemotherapy with EBRT. Of those receiving concurrent chemoradiation, patients most commonly received doxorubicin (n = 9). Two patients (7.1%) received targeted therapy; one received bevacizumab and one received sorafenib.

The median number of EBRT courses was 1 (range, 0–4), and the thyroid/thyroid bed was most commonly targeted (76.5%) (Supplementary Table 1). Other sites targeted by RT included left neck (61.8%), right neck (64.7%), mediastinum (17.6%), and metastases (20.6%). Of the patients who received EBRT to locoregional sites (thyroid, thyroid bed, and/or neck), the median cumulative dose was 3,000 cGy (IQR: 2,100–3,880) and median number of fractions was 16 (IQR: 10–24) (Supplementary Table 2). Locoregional EBRT was completed in 15 patients and discontinued early in 4 patients due to toxicity (n = 2 radiation toxicity, n = 1 chemotherapy toxicity; n = 1 postop complication). Some patients experienced more than one toxicity. Of the 2 patients who discontinued EBRT early due to radiation-specific toxicities, 1 was treated with IMRT and the other was treated with 2D/3D conformal RT. Other radiation toxicities that occurred were fatigue, mucositis, hoarseness, esophagitis, stridor, dermatitis, and neck edema. Of the 12 patients treated with IMRT, 7 (58.3%) had no reported toxicities, 2 (16.7%) had Grade 1 toxicities, and 3 (25.0%) had Grade 3 toxicities. Of the 7 patients treated with 2D/3D conformal RT, 3 (42.9%) had Grade 1 toxicities, 1 (14.3%) had Grade 2 toxicity, and 3 (42.9%) had Grade 3 toxicities. The in-field recurrence rate was 24.1% and EBRT was associated with improved recurrence-free survival (Supplementary Figure 1).

16 patients (57.1%) were never disease free, 11 patients (39.3%) had disease recurrence, and 1 patient (3.6%) had no evidence of disease progression throughout the study period. A majority of patients (n=20; 71.4%) died from thyroid cancer. Other causes of death included treatment toxicity (7.1%), other reasons (3.6%), and unknown (10.7%). Patients receiving surgery, EBRT, and chemotherapy had the best overall survival (Supplementary Figure 2). Pathology slides from 3 of the 5 patients with survival > 1 year were available for review, and a diagnosis of ATC was confirmed in all 3 cases.

**Figure 2:**
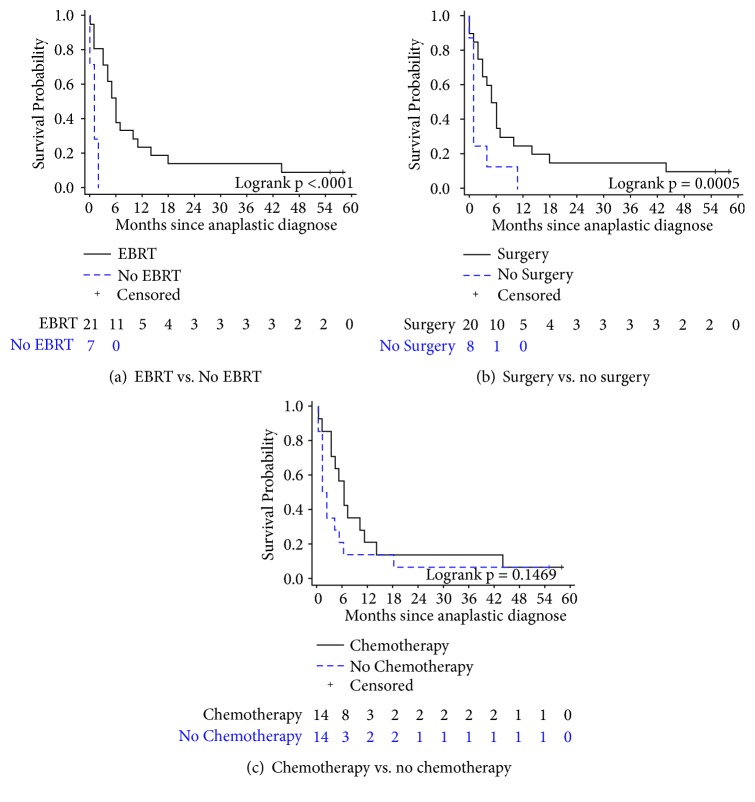
Overall survival for patients who underwent (a) EBRT versus no EBRT, (b) surgery versus no surgery, and (c) chemotherapy versus no chemotherapy.

For all patients, median OS was 4 months (95% CI: 1–6 months), with a 1-year survival rate of 17.9%. Median OS after completing the first course of EBRT was 6 months (95% CI: 3–10 months), with a 1-year survival rate of 23.8% (95% CI: 8.7–43.1%) as compared to a median OS of 1 month (95% CI: 0–2 months) and 0.0% 1-year survival rate in patients who did not receive EBRT (Figure 2(a)). Median OS after first surgical resection was 5.5 months (95% CI: 2–10 months), with a 1-year survival rate of 25.0% (95% CI: 9.1–44.9%) as compared to a median OS of 1 month (95% CI: 0–4 months) and 0.0% 1-year survival rate in patients who did not undergo surgery (Figure 2(b)). Patients receiving both surgery and EBRT had significantly better survival than those who received EBRT or surgery alone (p<0.0001 and p=0.0005, respectively). Median OS after receiving chemotherapy was 6 months (95% CI: 3–11 months), with a 1-year survival rate of 21.4% (95% CI: 5.2–44.8%). There was no significant difference in survival between patients who did and did not receive chemotherapy (p=0.15). In patients who received EBRT, IMRT was associated with a greater but not statistically significant 1-year survival rate (33.3%; 95% CI: 10.3–58.8%) as compared to 2D/3D conformal RT (11.1%; 95% CI: 0.6–38.8%). Curative intent RT also was associated with higher 1-year survival rate (42.9%; 95% CI: 9.8–73.4%) as compared to palliative RT (14.3%; 95% CI: 2.3–36.6%; p=0.0001).

Univariate (Table 3) and multivariate analyses (Table 4) were conducted to assess contributors to OS. Univariate analysis showed that stage at presentation, total radiation dose (<4,000 cGy vs. ≥4,000 cGy), fractionation scheme (1 vs. 2 fractions/day), receipt of chemotherapy, presence of initial or concomitant differentiated thyroid cancer diagnosis, lymphovascular invasion, extrathyroidal extension, and leukocytosis were not associated with OS (p>0.05). Age, EBRT, and surgery were associated with OS (p<0.05) and were used for multivariate analysis. Receipt of chemotherapy was also incorporated in the multivariate analysis to include all treatment modalities. The multivariate analysis showed that older age at diagnosis (HR: 1.079; 95% CI: 1.022–1.139; p=0.006) was associated with worse OS, while receipt of EBRT (HR: 0.174; 95% CI: 0.050–0.613; p=0.007) and surgery (HR: 0.198; 95% CI: 0.065–0.598; p=0.004) were associated with improved OS. Receipt of chemotherapy was not associated with OS on multivariate analysis (HR: 0.668; 95% CI: 0.274–1.633; p=0.38).

## 4. Discussion

The published literature on treatment of ATC consists mostly of single-institution retrospective studies with some larger studies and one meta-analysis [17–25]. They agree with the poor prognosis of ATC, encourage consideration of stage and prognostic factors for treatment recommendations, and indicate that a combination of surgery, radiotherapy, and chemotherapy is the most effective treatment regimen for anaplastic thyroid cancer. This study confirms that multimodality treatment leads to improved overall survival, with surgery and radiation therapy serving as the most important aspects of the treatment regimen. Chemotherapy did not provide a significant contribution to survival, corroborating prior studies [14, 26]. Interestingly, among variables incorporated in this analysis, receipt of EBRT was a significant predictor of overall survival. Receipt of EBRT also improved recurrence-free survival. Several studies have suggested that higher EBRT dose (≥40 Gy) is associated with prolonged median survival [14–16], however the lack of randomized prospective studies limits conclusions about the optimal fractionation scheme. One study supported hyperfractionation (46 Gy in 29 fractions) after observing improved local control [27], while others have argued against hyperfractionation due to the absence of survival benefit and increased incidence of toxicities, especially myelopathy [28, 29]. This study did not show a survival benefit from higher EBRT dose (≥40 Gy) or hyperfractionation. A recent preclinical study using an orthotopic mouse model of anaplastic thyroid cancer suggests that hypofractionation may be superior for tumor control and overall survival [30]. The optimal radiation dosing and fractionation plan remains unclear, emphasizing the need for prospective multi-institutional trials to investigate EBRT in anaplastic thyroid cancer patients.

The development of IMRT has been advantageous for head and neck cancer treatment, as it facilitates reduction of dose to nearby normal structures, specifically the spinal cord [5]. The patients in the current study experienced minimal radiation-specific toxicities and infrequently discontinued radiation treatment due to toxicity, possibly due to the increased use of IMRT treatments in this population. Although IMRT has been widely accepted as the radiation technique of choice for treatment of head and neck cancers [31], there is a paucity of published evidence supporting the treatment benefit of IMRT specifically in anaplastic thyroid cancer patients [32]. This study demonstrates an association between IMRT and improved 1-year survival relative to 2D/3D RT, which could be due to superior radiation techniques or better supportive care availability.

While curative radiation therapy is designed to treat all gross and microscopic disease to a dose expected to be lethal to the tumor cells, palliative radiation therapy with smaller treatment fields and/or lower dose may be more appropriate for some patients with distressing symptoms and poor functional status caused by their tumor burden. Because of the potential for severe morbidity from both disease invasion and treatment side effects in ATC, curative versus palliative treatment intent should be considered and discussed with the patient. Current guidelines, based off the AJCC TNM 7^th^ edition staging classification, recommend for Stage IVA patients to be treated curatively and Stage IVC patients to be treated palliatively with exceptions based on personalized patient scenarios [7]. However, in the current study, the majority of patients presented as AJCC TNM 8^th^ Stage IVB (1 of whom would be Stage IVA based on the AJCC TNM 7^th^ edition staging classification), for which guidelines recommend curative versus palliative therapy depending on the resectability of the primary tumor. Treatment intent for Stage IVB patients is a complex decision and should be discussed with a multidisciplinary team and the patient, including consideration of the patient's quality of life. This study showed that curative RT intent corresponded with improved survival as compared to palliative intent. This may be related to patient functional status impacting the treating physician's intent and/or improved outcomes with more aggressive therapeutic approach. Despite the caveat that patients with smaller disease burden and better functional status are more likely to undergo curative treatment in the first place, this finding emphasizes the importance of considering treatment intent in selecting a treatment approach, particularly for Stage IVB patients given that one patient in our study was up-staged from IVA to IVB using the AJCC TNM 8^th^ edition staging classification. Thus, we propose that Stage IVB patients with favorable prognostic factors, including younger age (<70 years old), anaplastic transformation from differentiated thyroid carcinoma, lesser disease extension, and smaller primary tumor size (< 5 cm), should be considered for curative intent therapy [18, 33].

While surgery and EBRT provide improved local control of anaplastic thyroid cancer, the aggressive nature of the disease commonly results in metastatic spread and death. Unfortunately, ATC is poorly responsive to chemotherapy [14, 26, 34]. Targeted therapy and immunotherapy may bring new opportunities for systemic treatment options. Many mutations, including BRAF, NRAS, TP53, HRAS, KRAS, PIK3CA, and RB1, have been identified as potential targets [35]. Several prospective trials have begun studying agents aimed at these mutations. Tyrosine kinase inhibitors, including axitinib, sorafenib, imatinib, and lenvatinib have been shown to cause partial response or stable disease in some ATC patients [36–39]. A phase II trial investigating dabrafenib, a BRAF inhibitor, in combination with trametinib, a MEK inhibitor, showed that this regimen caused a 69% response rate with minimal toxicities [40]. Additionally, case reports have shown initial tumor response from vemurafenib [41], erlotinib [42], and neoadjuvant valproic acid [5, 43]. Novel systemic therapies should also be considered for reducing morbidity in patients being treated palliatively or in patients with acute disease progression. In our study, the two patients who received targeted therapy (bevacizumab or sorafenib) did so under palliative conditions. Even though both of these patients had disease progression while on targeted therapy, they both experienced minimal drug toxicity and had a greater than average overall survival (>4 months) following their ATC diagnosis. Additionally, two reports have shown that the use of a selective BRAF inhibitor was effective in preventing tracheostomy placement after providing rapid relief in patients with impending airway compromise [44]. Overall, further development of systemic therapies should be investigated to improve survival and reduce morbidity in this disease.

This study has several limitations. First, it is a retrospective review, which is prone to bias, misclassification, and measurement error. Second, only 36 patients with ATC were identified at our institution over a 25-year time period, and 8 of these patients had insufficient information in their medical record. Thus, this study is limited by a small number of subjects and poor statistical power. Furthermore, patient performance status was often not readily identifiable in the medical record. Although the multivariate analysis adjusted for factors related to disease prognosis, there were likely unmeasured factors, such as patient performance status, that were related to both treatment decisions and outcomes. Finally, a majority of patients were never disease free through the study time period, producing underestimations for recurrence rate and difficulty interpreting local control. These limitations further emphasize the need for multicenter prospective studies to investigate ATC treatment options in order to identify the most favorable multimodality approach.

## 5. Conclusions

Despite the limitations in this study and others, several themes have emerged. Surgical resection with EBRT is the most effective modality for local control and has been associated with improved survival. Administering a higher EBRT dose by IMRT is preferred [18], but the optimal RT fractionation scheme remains unclear. Chemotherapy is primarily used for radiosensitization, and the development of more effective systemic therapies is necessary. Inclusion of anaplastic thyroid cancer patients in clinical trials involving targeted and immunotherapies will be helpful for further understanding systemic treatment options. Until better systemic therapies are developed and refined for anaplastic thyroid cancer, likelihood of cure for patients with this aggressive disease remains low, particularly when presenting with later stages of disease.

## Figures and Tables

**Table 1:** Patient characteristics.

	All patients (N=28)
Age at diagnosis (years)	
Median (IQR)	70.9 (63.8 - 74.7)

Sex	
Female	16 (57.1%)
Male	12 (42.9%)

Race	
Caucasian	22 (78.6%)
African-American	6 (21.4%)

Leukocytosis	
No	12 (42.9%)
Yes	12 (42.9%)
Unknown	4 (14.3%)

Initial or concomitant diagnosis of differentiated thyroid cancer (papillary or follicular)	
No	22 (78.6%)
Yes	6 (21.4%)

Stage	
IVA	2 (7.1%)
IVB	20 (71.4%)
IVC	5 (17.9%)
Unknown	1 (3.6%)

T stage	
T2	1 (3.6%)
T3	1 (3.6%)
T4a	13 (46.4%)
T4b	12 (42.9%)
Unknown	1 (3.6%)

N stage	
N0	9 (32.1%)
N1a	1 (3.6%)
N1b	4 (14.3%)
Nx or Unknown	14 (50.0%)

M stage	
M0	7 (25.0%)
M1	5 (17.9%)
Mx or Unknown	16 (57.1%)

Lymphovascular invasion	
No	5 (17.9%)
Yes	14 (50.0%)
Unknown	9 (32.1%)

Extrathyroidal extension	
No	4 (14.3%)
Yes	23 (82.1%)
Unknown	1 (3.6%)

Counts and column percentages are presented unless otherwise specified.

**Table 2:** Surgical, radioactive iodine, radiotherapy, and systemic treatment characteristics.

	All patients (N=28)
Surgery	
No	8 (28.6%)
Yes	20 (71.4%)

Number of surgeries per patient	
0	8 (28.6%)
1	14 (50.0%)
2	4 (14.3%)
4	2 (7.1%)

Type(s) of surgery	
Lobectomy	7 (25.0%)
Thyroidectomy	12 (42.9%)
Lymph node dissection	9 (32.1%)
Metastasectomy	1 (3.6%)

Radioactive iodine	
No	24 (85.7%)
Yes, after ATC diagnosis	2 (7.1%)
Yes, before ATC diagnosis	2 (7.1%)

EBRT to any site	
No	7 (25.0%)
Yes	21 (75.0%)

EBRT to thyroid bed/neck	
No	9 (32.1%)
Yes	19 (67.9%)

Total radiation dose (cGy)	
< 4,000	11 (39.3%)
≥ 4,000	6 (21.4%)
Unknown	4 (14.3%)

Radiation fractionation (fractions/day)	
1	8 (28.6%)
> 1	9 (32.1%)
Unknown	4 (14.3%)

More than one EBRT treatment course	
No	19 (67.9%)
Yes	9 (32.1%)

EBRT initial intent	
No EBRT	7 (25.0%)
Palliative	14 (50.0%)
Curative	7 (25.0%)

EBRT initial technique	
No EBRT	7 (25.0%)
2D	4 (14.3%)
3D	3 (10.7%)
IMRT/VMAT	12 (42.9%)
Unknown	2 (7.1%)

Systemic therapy	
No	14 (50.0%)
Yes	14 (50.0%)

Type(s) of chemotherapy used with EBRT	
Doxorubicin	4 (14.3%)
Doxorubicin, Cisplatin	2 (7.1%)
Doxorubicin, Carboplatin, Paclitaxel	1 (3.6%)
Doxorubicin, Carboplatin, Paclitaxel, Cisplatin, Pemetrexed, Bevacizumab	1 (3.6%)
Doxorubicin, Carboplatin, Paclitaxel, Cyclophosphamide, Vinorelbine, Gemcitabine	1 (3.6%)
Carboplatin, Paclitaxel	1 (3.6%)
Sorafenib	1 (3.6%)

All treatments	
Surgery + EBRT + Chemotherapy	10 (35.7%)
Surgery + EBRT	5 (17.9%)
EBRT + Chemotherapy	3 (10.7%)
EBRT only	3 (10.7%)
Surgery only	5 (17.9%)
Chemotherapy only	1 (3.6%)
No treatment	1 (3.6%)

Counts and column percentages are presented unless otherwise specified.

EBRT = external beam radiation therapy; IMRT = intensity modulated radiation therapy; VMAT = volumetric modulated arc therapy.

**Table 3:** Univariate overall survival analysis (N=28, # events=26, 7% censored).

	N	Deaths	HR (95% CI)	P-Value
Age at ATC diagnosis (years)	28		1.064 (1.014 - 1.116)	0.011

Stage				0.18
IVA	2	1 (50.0%)	Reference	
IVB	20	19 (95.0%)	3.771 (0.499 - 28.468)	
IVC	5	5 (100.0%)	7.246 (0.804 - 65.269)	

Receipt of EBRT				0.002
No EBRT	7	7 (100.0%)	Reference	
EBRT	21	19 (90.5%)	0.133 (0.038 - 0.463)	

Total locoregional radiation dose (cGy)				0.32
< 4,000	11	9 (81.8%)	Reference	
≥ 4,000	6	6 (100.0%)	1.799 (0.568 - 5.700)	

Radiation fractionation (fractions/day)				0.12
1	8	7 (87.5%)	Reference	
2	9	8 (88.9%)	0.427 (0.147 - 1.235)	

Receipt of surgery				0.036
No surgery	8	8 (100.0%)	Reference	
Surgery	20	18 (90.0%)	0.384 (0.157 - 0.938)	

Receipt of chemotherapy				0.21
No chemotherapy	14	13 (92.9%)	Reference	
Chemotherapy	14	13 (92.9%)	0.605 (0.276 - 1.323)	

Initial or concomitant diagnosis of differentiated thyroid cancer (papillary or follicular)				0.31
No	22	21 (95.5%)	Reference	
Yes	6	5 (83.3%)	0.602 (0.224 - 1.617)	

Lymphovascular invasion				0.76
No	5	5 (100.0%)	Reference	
Yes	14	12 (85.7%)	0.846 (0.287 - 2.491)	

Extrathyroidal extension				0.21
No	4	3 (75.0%)	Reference	
Yes	23	22 (95.7%)	2.182 (0.649 - 7.340)	

Leukocytosis				0.12
No	12	11 (91.7%)	Reference	
Yes	12	11 (91.7%)	1.994 (0.833 - 4.773)	

Patients with unknown values for a covariate were excluded from the respective univariate analysis.

Counts and row percentages of deaths are presented for all covariates except for age.

Hazard ratios and confidence intervals are from Cox proportional hazards models, with p-values calculated by Wald chi-square tests.

HR = hazard ratio; CI = confidence interval; EBRT = external beam radiation therapy.

**Table 4:** Multivariate overall survival analysis (N=28, # events=26, 7% censored).

	HR (95% CI)	P-Value
Age at ATC diagnosis (years)	1.079 (1.022 -1.139)	0.006

Receipt of EBRT		
No EBRT	Reference	
EBRT	0.174 (0.050 - 0.613)	0.007

Receipt of surgery		
No surgery	Reference	
Surgery	0.198 (0.065 - 0.598)	0.004

Receipt of chemotherapy		
No chemotherapy	Reference	
Chemotherapy	0.668 (0.274 - 1.633)	0.38

Hazard ratios and confidence intervals are from a Cox proportional hazards model, with p-values calculated by a Wald chi-square test.

HR = hazard ratio; CI = confidence interval; EBRT = external beam radiation therapy.

## Data Availability

The clinical data used to support the findings of this study are included within the article and within the supplementary information files.

## Supplementary Materials

Supplementary Materials
* Supplementary Table 1* describes treatment characteristics for patients who underwent EBRT to any body site.* Supplementary Table 2* describes treatment characteristics for patients who underwent EBRT to the thyroid/thyroid bed/neck.* Supplementary Figure 1* shows the recurrence-free survival for patients who underwent EBRT versus no EBRT.* Supplementary Figure 2* shows the overall survival for all patients according to treatment regimen.
