# Supplementary material for: Treatment Outcomes in Anaplastic Thyroid Cancer
Source: J Thyroid Res. 2019 May 23;2019:8218949. doi: 10.1155/2019/8218949 (PMC6556325; doi:10.1155/2019/8218949)
Supplement: Supplementary Materials — Supplementary Table 1 describes treatment characteristics for patients who underwent EBRT to any body site. Supplementary Table 2 describes treatment characteristics for patients who underwent EBRT to the thyroid/thyroid bed/neck. Supplementary Figure 1 shows the recurrence-free survival for patients who underwent EBRT versus no EBRT. Supplementary Figure 2 shows the overall survival for all patients according to treatment regimen. [file 8218949.f1.pdf]

SUPPLEMENTARY MATERIALS

Supplementary Table 1: Treatment characteristics for all patients who underwent EBRT

	All courses (N=34)	First course (N=21)	Second course (N=9)	Third course (N=3)	Fourth course (N=1)
EBRT intent					
Curative	11 (32.4%)	7 (33.3%)	3 (33.3%)	1 (33.3%)	0 (0.0%)
Palliative	23 (67.6%)	14 (66.7%)	6 (66.7%)	2 (66.7%)	1 (100.0%)
EBRT type					
2D	10 (29.4%)	5 (23.8%)	4 (44.4%)	1 (33.3%)	0 (0.0%)
3D conformal	3 (8.8%)	3 (14.3%)	0 (0.0%)	0 (0.0%)	0 (0.0%)
IMRT/VMAT	17 (50.0%)	11 (52.4%)	5 (55.6%)	1 (33.3%)	0 (0.0%)
SBRT	1 (2.9%)	0 (0.0%)	0 (0.0%)	1 (33.3%)	0 (0.0%)
SRS	1 (2.9%)	0 (0.0%)	0 (0.0%)	0 (0.0%)	1 (100.0%)
Unknown	2 (5.9%)	2 (9.5%)	0 (0.0%)	0 (0.0%)	0 (0.0%)
Site(s) of treatment					
Thyroid/Thyroid bed	26 (76.5%)	18 (85.7%)	6 (66.7%)	2 (66.7%)	0 (0.0%)
Left neck	21 (61.8%)	16 (76.2%)	4 (44.4%)	1 (33.3%)	0 (0.0%)
Right neck	22 (64.7%)	16 (76.2%)	5 (55.6%)	1 (33.3%)	0 (0.0%)
Mediastinum	6 (17.6%)	3 (14.3%)	3 (33.3%)	0 (0.0%)	0 (0.0%)
Metastases	7 (20.6%)	2 (9.5%)	3 (33.3%)	1 (33.3%)	1 (100.0%)
Other	2 (5.9%)	1 (4.8%)	1 (11.1%)	0 (0.0%)	0 (0.0%)
Number of fractions					
Median (IQR)	14.0 (10.0 - 23.0)	17.0 (11.0 - 27.0)	10.0 (10.0 - 17.0)	11.0 (3.0 - 18.0)	1.0 (1.0 - 1.0)
Total dose (cGy)					
Median (IQR)	3000.0 (2100.0 - 3880.0)	3500.0 (2500.0 - 4500.0)	2690.0 (1750.0 - 3000.0)	2000.0 (1800.0 - 3600.0)	5900.0 (5900.0 - 5900.0)
RT course completion					
Completed	27 (79.4%)	15 (71.4%)	8 (88.9%)	3 (100.0%)	1 (100.0%)

	All courses (N=34)	First course (N=21)	Second course (N=9)	Third course (N=3)	Fourth course (N=1)
Discontinued early due to toxicity	3 (8.8%)	3 (14.3%)	0 (0.0%)	0 (0.0%)	0 (0.0%)
Discontinued early due to other reason	2 (5.9%)	2 (9.5%)	0 (0.0%)	0 (0.0%)	0 (0.0%)
In-field recurrence					
No	23 (67.6%)	14 (66.7%)	5 (55.6%)	3 (100.0%)	1 (100.0%)
Yes	8 (23.5%)	6 (28.6%)	2 (22.2%)	0 (0.0%)	0 (0.0%)
Unknown	2 (5.9%)	0 (0.0%)	2 (22.2%)	0 (0.0%)	0 (0.0%)

Supplementary Table 2. Treatment characteristics for patients who underwent EBRT to the thyroid/thyroid bed/neck

	All courses (N=29)	First course (N=19)	Second course (N=8)	Third course (N=2)
EBRT intent				
Curative	10 (34.5%)	7 (36.8%)	3 (37.5%)	0 (0.0%)
Palliative	19 (65.5%)	12 (63.2%)	5 (62.5%)	2 (100.0%)
EBRT type				
2D	9 (31.0%)	5 (26.3%)	3 (37.5%)	1 (50.0%)
3D conformal	3 (10.3%)	3 (15.8%)	0 (0.0%)	0 (0.0%)
IMRT/VMAT	15 (51.7%)	9 (47.4%)	5 (62.5%)	1 (50.0%)
Unknown	2 (6.9%)	2 (10.5%)	0 (0.0%)	0 (0.0%)
Site(s) of treatment				
Thyroid/Thyroid bed	26 (89.7%)	18 (94.7%)	6 (75.0%)	2 (100.0%)
Left neck	21 (72.4%)	16 (84.2%)	4 (50.0%)	1 (50.0%)
Right neck	22 (75.9%)	16 (84.2%)	5 (62.5%)	1 (50.0%)
Number of fractions				
Median (IQR)	16.0 (10.0 - 24.0)	19.0 (12.0 - 30.0)	10.0 (10.0 - 20.0)	14.5 (11.0 - 18.0)

	All courses (N=29)	First course (N=19)	Second course (N=8)	Third course (N=2)
Total dose (cGy)				
Median (IQR)	3000.0 (2100.0 - 3880.0)	3550.0 (2450.0 - 4500.0)	2380.0 (1500.0 - 3000.0)	2800.0 (2000.0 - 3600.0)
RT course completion				
Completed	22 (75.9%)	13 (68.4%)	7 (87.5%)	2 (100.0%)
Discontinued early due to toxicity	3 (10.3%)	3 (15.8%)	0 (0.0%)	0 (0.0%)
Discontinued early due to other reason	2 (6.9%)	2 (10.5%)	0 (0.0%)	0 (0.0%)
In-field recurrence				
No	20 (69.0%)	13 (68.4%)	5 (62.5%)	2 (100.0%)
Yes	7 (24.1%)	5 (26.3%)	2 (25.0%)	0 (0.0%)
Unknown	1 (3.4%)	0 (0.0%)	1 (12.5%)	0 (0.0%)

Counts and column percentages are presented unless otherwise specified.
EBRT = External beam radiation therapy; IMRT = Intensity modulated radiation therapy; VMAT = Volumetric modulated arc therapy; RT = Radiotherapy.

Supplementary Figure 1: Recurrence-free survival for patients who underwent EBRT versus no EBRT

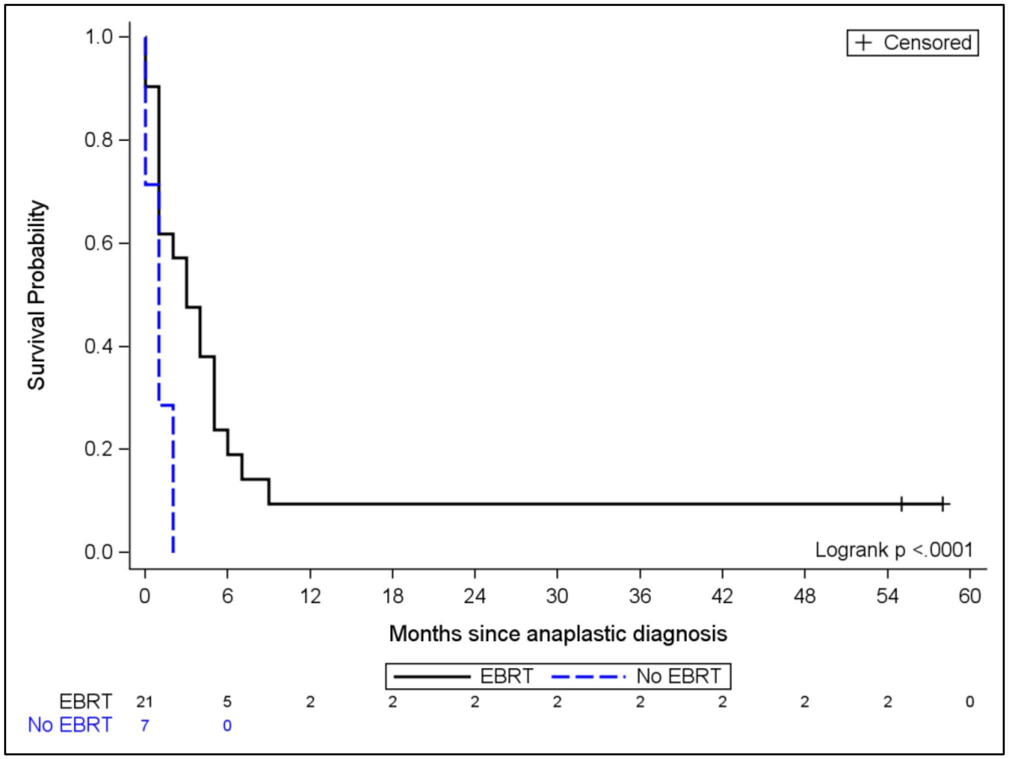

	Total	Events	Censored	1-Year Survival Rate (95% CI)	Median Survival (months) (95% CI)
EBRT	21	19 (90%)	2 (10%)	0.095 (0.016 - 0.261)	3.0 (1.0 - 5.0)
No EBRT	7	7 (100%)	0 (0%)		1.0 (0.0 - 2.0)
Total	28	26 (93%)	2 (7%)		

Supplementary Figure 2: Overall survival for all patients according to treatment regimen

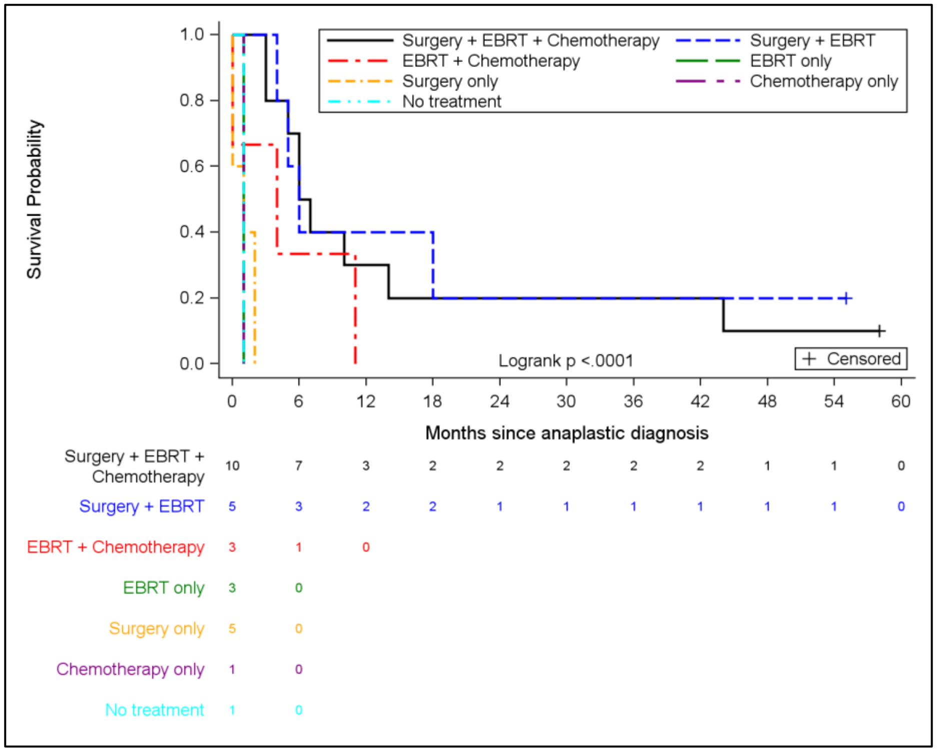

	Total	Deaths	Censored	1-Year Survival Rate (95% CI)	Median Survival (months) (95% CI)
Surgery + EBRT + Chemotherapy	10	9 (90%)	1 (10%)	0.300 (0.071 - 0.578)	6.5 (3.0 - 14.0)
Surgery + EBRT	5	4 (80%)	1 (20%)	0.400 (0.052 - 0.753)	6.0 (4.0 - NE)
EBRT + Chemotherapy	3	3 (100%)	0 (0%)		4.0 (0.0 - 11.0)
EBRT only	3	3 (100%)	0 (0%)		1.0 (NE - NE)
Surgery only	5	5 (100%)	0 (0%)		1.0 (0.0 - 2.0)
Chemotherapy only	1	1 (100%)	0 (0%)		1.0 (NE - NE)
No treatment	1	1 (100%)	0 (0%)		1.0 (NE - NE)
Total	28	26 (93%)	2 (7%)		

NE = Non-evaluable
